# Molecular Characterization of a New *Moniliformis* sp. From a Plateau Zokor (*Eospalax fontanierii baileyi*) in China

**DOI:** 10.3389/fmicb.2022.806882

**Published:** 2022-03-09

**Authors:** Guo-Dong Dai, Hong-Bin Yan, Li Li, Lin-Sheng Zhang, Zhan-Long Liu, Sheng-Zhi Gao, John Asekhaen Ohiolei, Yao-Dong Wu, Ai-Min Guo, Bao-Quan Fu, Wan-Zhong Jia

**Affiliations:** ^1^State Key Laboratory of Veterinary Etiological Biology, National Professional Laboratory for Animal Echinococcosis, Key Laboratory of Veterinary Parasitology of Gansu Province, Key Laboratory of Zoonoses of Agriculture Ministry, Lanzhou Veterinary Research Institute, Chinese Academy of Agricultural Sciences, Lanzhou, China; ^2^Xiahe Animal Centre for Disease Control and Prevention, Xiahe, China; ^3^Animal Centre for Disease Control and Prevention, Lanzhou, China; ^4^Jiangsu Co-innovation Centre for Prevention and Control of Important Animal Infectious Disease, Yangzhou, China

**Keywords:** *Moniliformis*, mitochondrial genome, 18S rDNA gene, gene annotation, phylogeny, *cox*1 gene

## Abstract

In the present study, a new species of the genus *Moniliformis* species is described taxonomically in the mitochondrial genomic context. The parasite was found in a plateau zokor captured in a high-altitude area of Xiahe County of Gansu Province, China. The mitochondrial (*mt*) genome length of this new species was 14,066 bp comprising 36 genes and 2 additional non-coding regions (SNR and LNR), without *atp*8. The molecular phylogeny inferred by the cytochrome c oxidase subunit I gene (*cox*1) and the18S ribosomal RNA gene (18S rDNA) sequences showed that the parasite as a sister species to other *Moniliformis* spp. and was named *Moniliformis* sp. XH-2020. The phylogeny of the concatenated amino acid sequences of the 12 protein-coding genes (PCGs) showed *Moniliformis* sp. XH-2020 in the same cluster as *Macracanthorhynchus hirudinaceus* and *Oncicola luehe*i confirming the *cox*1 and 18S rDNA phylogenetic inference. In addition, the entire *mt* genome sequenced in this study represents the first in the order Moniliformida, providing molecular material for further study of the phylogeny of the class Archiacanthocephala. Moreover, the species of this class, use arthropods as intermediate hosts and mammals as definitive hosts and are agents of acanthocephaliasis, a zoonosis in humans. Therefore, this study not only expands the host range among potential wild animal hosts for Archiacanthocephalans which is of great ecological and evolutionary significance but also has important significance for the research of zoonotic parasitic diseases.

## Introduction

Acanthocephalans are obligate endoparasites of vertebrates that complete their life cycle with the participation of arthropods (Kennedy, 2006). At present, there are 1,330 verified acanthocephalans across four classes. *Moniliformis* sp. XH-2020 identified in this study is within the order Moniliformida Schmidt (1972). So far, about 20 species exist in the order Moniliformida (Amin, 2013), its main definitive and intermediate hosts are rodents and insects respectively. Carnivores such as the families Canidae, Felidae and Mustelidae (Kozlov, 1977), as well as birds and hedgehogs (Khaldi et al., 2012), can serve as facultative definitive hosts. Human cases of infection have been reported (Berenji et al., 2007). The parasite develops into an adult and completes its life cycle only after the intermediate host of the infected larva is preyed on by the definitive host (Khalaf et al., 2021). *Moniliformis* sp. XH-2020 was found in the small intestine of the plateau zokor, *Eospalax fontanierii baileyi* Thomas, 1911 (Rodentia: Spalacidae), which is a typical subterranean rodent (Su et al., 2015, 2018). *Eospalax fontanierii* mainly inhabits high altitude areas like the Qinghai-Tibet Plateau (average elevation 4,500 m) and southwestern part of Gansu province (average elevation 2,500 m). The plateau region has a special ecological environment with less anthropogenic activities, therefore, many parasites maintain their life cycle by taking advantage of the predator-prey relationship between wild animals.

Taxonomically, *Moniliformis* sp. XH-2020 belongs to Acanthocephala; Archiacanthocephala; Moniliformida; Moniliformidae. However, the taxonomic classification of acanthocephalans has not been completely resolved due to the limited species and genetic information available. Moreover, accurate classification of acanthocephalans based on morphology and ecology alone remains a major challenge (Amin, 2013). In particular, the monophyletic or paraphyletic issues of Palaeacanthocephala and the phylogenetic position of Polyacanthocephala based on nuclear ribosomal DNA remains unresolved (Gazi et al., 2016). Interestingly, studies on *mt* genome have demonstrated its potentials in resolving taxonomic challenges due to the high mutation rate, maternal inheritance, and highly conserved characteristics (Jia et al., 2012).

There are very few studies on Moniliformidae parasitism in rodents in China. Elsewhere, more studies continue to report new species of acanthocephalans (Amin et al., 2016, 2019; Guerreiro Martins et al., 2017; Gomes et al., 2020; Lynggaard et al., 2021) indicating a high species richness. However, no complete mitochondrial (*mt*) genome sequence is available for this order, hence, besides analysis of the taxonomic status of *Moniliformis* spp. and description of the mitochondrial genomic characteristic we also provide the complete *mt* genome sequence while investigating the presence of *Moniliformis* spp. in plateau zokors captured in the high altitude areas of Gansu province. This study further expands the definitive host range of Moniliformidae in China as well as the growing number of acanthocephalans, which has reference value for the control of acanthocephaliasis. Meanwhile, the complete mitochondrial genome obtained in this study provides molecular material for further molecular evolutionary analysis of *Moniliformis* spp.

## Materials and Methods

### Study Area and Parasite Materials

A total of 1,426 plateau zokors (*Eospalax fontanierii baileyi*) were caught by mousetraps in Xiahe County (102°52′ E; 35°2′ N; altitude at 3,105 m), Luqu County (102°58′ E; 34°48′ N; altitude at 3,114 m) and Hezuo City (103°22′ E; 35°18′ N; altitude at 2,968 m) in Gansu Province, China. This was achieved during the population control program that is conducted annually to protect the grassland ecology from the destructive impact of rodents on grassland vegetation. After trapping, carcasses were dissected and examined based on common predilection site of helminth parasites (including the liver, lungs, intestines, thoracic cavity, and abdomen). The organs and bodies were then disposed according to local guidelines and regulations. Encountered toruliform parasites were cleaned using phosphate buffer saline and preserved in 70% alcohol for identification.

### Morphological Feature

The specimens were collected as they fell off the intestinal wall during the examination of the intestinal content and fixed in 70% alcohol. This was followed by morphological identification of the worms. In particular, the size, color, shape, nodal and somatic segments of the worm were observed. For scanning electron microscopy (SEM), specimens previously fixed in 70% ethanol were dehydrated in an ascending ethanol series (80, 90, 100%). Then the scolex and tail of the parasite were cut off and were processed for SEM following standard methods, including critical point-dried (CPD) with CO_2_ in sample baskets, mounted with silver adhesive tape on aluminum stubs and sputter-coated with a 20 nm layer of gold for 3 min using a Polaron #3500 sputter coater. Samples were then examined using an Apreo S microscope (Apreo S SEM, Thermo Scientific, United States) under low vacuum conditions using 20 Kv at the Electron Microscopy Centre of Lanzhou University. Finally, digital images were stored on a USB for further use.

### Extraction, Primer Design, Amplification and Sequencing

Host liver and worm segments were selected for genomic DNA (gDNA) extraction, and the specific operation steps were carried out according to the manufacturer’s instructions (DNeasy Blood & Tissue Kit, Germany). The 18S rDNA gene, *cox*1 gene, the complete *mt* genome of the acanthocephalans and plateau zokor’s *cox*1 gene were downloaded from NCBI GenBank database^1^ to serve as reference sequences (Supplementary Table 1). Using Oligo 6.0, primers targeting the *cox*1 gene of the host were designed to identify the zokor species while the 18S rDNA was used to identify the parasite specimens. PCR was performed in a final reaction volume of 50 μl (HIQ Pfu Master Mix polymerase 25 μl, ddH_2_O 19 μl, forward primers 2 μl, reverse primers 2 μl, gDNA template 2 μl) using the following amplification program: predenaturation at 98°C for 3 min, denaturation at 98°C for 10 sec, annealing at 56°C for 20 sec, extension at 72°C for 70 sec, final extension at 72°C for 5 min. All PCR products were visualized in 1% agarose gel electrophoresis and sequenced for BLASTn. The BLASTn result of the parasite specimen demonstrated similarity to the order Archiacanthocephala that comprised two species (*M. hirudinaceus* and *O. luehei*) with complete *mt* genome sequences, which were used as reference sequences to design 9 overlapping primers (Table 1) to amplify the complete *mt* genome sequences of the current specimen. All primers in this study were synthesized by Tsingke Biological Technology Company (Xi’an, China).

**TABLE 1:** List of primer pairs used in the amplification of the complete mitochondrial genome and gene of the parasite and the host’s *cox*1 gene.

Primer names		Primer pairs (5′–3′)	Position (starting from *cox*1)
*mt*DNA1	F	GTGCTTCGGTGGGTGTATTCTACT	1-1582
	R	TGATTACGCTACCTTAGCACAGTC	
*mt*DNA2	F	ACTTAGCTCGGTTGAGAGGTGGGC	1420-2592
	R	TCTGGCTCACACCGATCTAAACTC	
*mt*DNA3	F	ATTTCATTGGGGTAATGGTTGAAGC	2389-4313
	R	AAACCACTCGTTAGCTCCGCGC	
*mt*DNA4	F	GGTGAATATACTGTAAATTTTCAAG	4194-6680
	R	GCCACCCTGAATGTAACTATCCTCC	
*mt*DNA5	F	CCTAAGGTTCATGTAGAGGCTTCT	5820-8860
	R	CGAACTAATGTAGAATCCCCTACCG	
*mt*DNA6	F	GGMTATGTTTTRCCTTGGGGGC	8709-10275
	R	TTAGTCTCTACAACACATACCATCC	
*mt*DNA7	F	ATACGGGGGTTGGCCCAAATGG	9859-11115
	R	AACCGCTGGCACGCTCTTAATCAAC	
*mt*DNA8	F	GTCATTTCAACGAATGAGCGTTG	10632-13257
	R	AGTAGACCAGCTAACTTATAAACCG	
*mt*DNA9	F	ATTGAGATCAGGAGTGACGGTGACC	12780-260
	R	AGTAGACCAGCTAACTTATAAACCG	
18S rDNA	F	ACCGCGATGGCTCATTACAT	
	R	TGTGTACAAAGGGCAGGGAC	
zokor *cox*1	F	GGWGCTTGAGCAGGMATAG	
	R	GGACATCCGTGAAGTCATTC	

### Sequence Assembly, Annotation and Analysis

The DNAstar software package was used to assemble the 9 overlapping sequence reads. Using the two reference species, we performed a preliminary *mt* genome annotation using the online program GeSeq^2^ (Tillich et al., 2017). The annotated results were further checked by SnapGene software, and manual modifications were carried out according to the reference sequences. The online tool ARWEN^3^ was used to predict tRNA genes (Laslett and Canback, 2008). VARNA software^4^ was used to draw the secondary structures of tRNA (Darty et al., 2009). Nucleic acid sequence, codon usage, amino acid composition and base ratio were all completed in MEGA7 (version 7.0.26) (Kumar et al., 2016).

### Phylogenetic Analysis

For the evolutionary analysis of the identified parasite specimen, this study used three gene datasets: the *cox*1 sequences of 61 acanthocephalans, the concatenated amino acid sequences of the 12 *mt* PCGs of 16 acanthocephalans and the 18S rDNA gene of 23 acanthocephalans, all of which represented the four classes (Archiacanthocephala, Palaeacanthocephala, Eoacanthocephala and Polyacanthocephala) (Muhammad et al., 2019). *Philodina citrina* Ehrenberg, 1832 (Rotifera: Philodina) was used as an outgroup. Sequence alignment was completed using MAFFT (Katoh and Standley, 2013). The software trimAl was used to remove poorly aligned regions from the alignment to improve the quality of subsequent analyses (Capella-Gutiérrez et al., 2009). DAMBE v. 7.2.136^5^ was used to verify the replacement saturation of the sequence after alignment (Xia, 2018). The phylogenetic analysis for three datasets was carried out using Bayesian Inference (BI), and Maximum-Likelihood (ML) method was further used to verify the phylogenetic results. For Bayesian Inference (BI) (Ronquist and Huelsenbeck, 2003), settings for the *cox*1 dataset were lser rates = gamma, prset aamodelpr = Mixed, mcmc ngen = 4,000,000, samplefreg = 1,000, nchains = 4; and for 18S rDNA gene and concatenated amino acid sequences of the12 PCGs, lser nst = 6, rates = invgamma, mcmc ngen = 1,000,000, samplefreg = 1,000, nchains = 4. The analysis continued until the average standard deviation of split frequencies was lower than 0.01. On completion, 25% were discarded as burn-in. The phylograms were viewed and embellished using the online tool: iTOL^6^ (Letunic and Bork, 2019). For Maximum-Likelihood (ML) inference, the online program IQ-TREE^7^ was used (Trifinopoulos et al., 2016).

## Results and Discussion

### Species Identification of Host and Parasite

**FIGURE 1:**
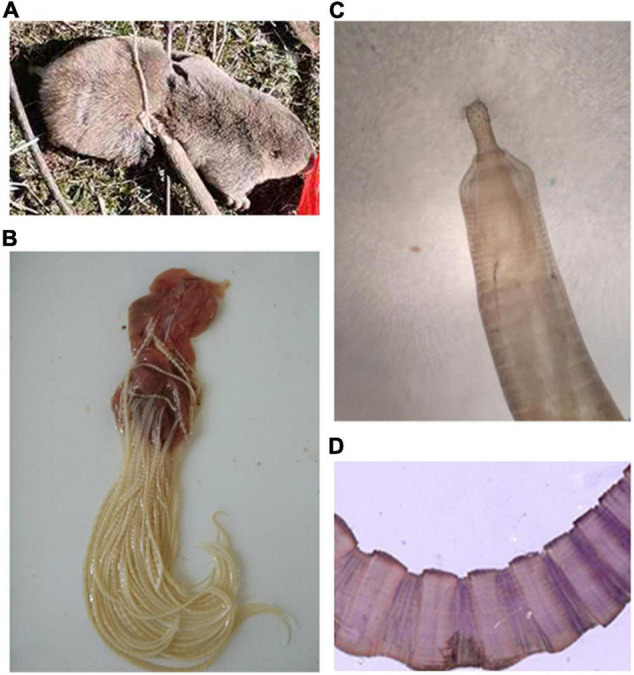
Morphological characteristics of the hosts and *Moniliformis* sp. XH-2020. **(A)** The captured plateau zokor. **(B)** Parasite adsorbed in the small intestinal wall after repeated washes with PBS buffer. **(C,D)** The structure of proboscis and segments of the parasite as seen under a light microscope (100×).

The plateau zokor has a thick and round body with a short snout, small eyes, short tail, and strong limbs. The adult coat is grayish-brown from head to tail, darker gray on the ventral surface than on the back, bluish-gray or dark gray on the juveniles and the semi-adults. Adult body length is about 160–235 mm and weighs about 173–490 g (Figure 1A). Three cases of infection were detected in 1,426 individuals (the infection rate was 0.21%) of the plateau zokor in the high elevation pastoral areas of Gansu, during May 2020. The thorny-headed worm identified in this study is closely related to species of the family Moniliformidae. The parasite is cylindrical and toruliform in shape, with light yellow coloration and measures 5–10 cm in length (Figure 1B). Light microscopy revealed that the parasite possessed a small proboscis with many hooks and toruliform segments (Figures 1C,D).

**FIGURE 2:**
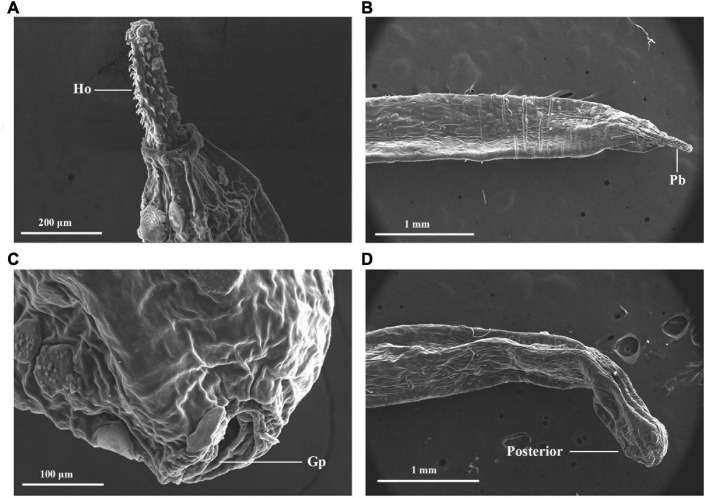
External morphology of *Moniliformis* sp. XH-2020 via SEM: **(A)** anterior end of the adult with proboscis; **(B)** lateral view of the proboscis with hooks, **(C)** posterior end of the adult, **(D)** posterior end showing a terminal gonopore. Pb, proboscis; Ho, hook; Gp, gonopore.

The SEM image of the anterior and posterior regions are shown in Figures 2A–D. Considering morphological limitations and overlapping characteristics among similar organisms, the host and parasite species were further investigated using genetic molecular markers.

In this study, the partial nucleotide sequences of the host *cox*1 gene (1,299 bp) and 18S rDNA gene (1,414 bp) of the parasite were used for identification. The BLASTn results confirmed the plateau zokor as *Eospalax fontanierii baileyi* with a 100% identity while the parasite demonstrated 98.87–100% identity with the genus *Moniliformis*, but the mitochondrial *cox*1 gene was only 76.12–87% similar to other acanthocephalans in GenBank. Therefore, in order to accurately describe the taxonomic status of the specimen, the reference sequences of *cox*1, 18S rDNA and the entire *mt* genome of other acanthocephalans were downloaded from GenBank followed by phylogenetic analysis.

### Features of the Mitochondrial Genome

**FIGURE 3:**
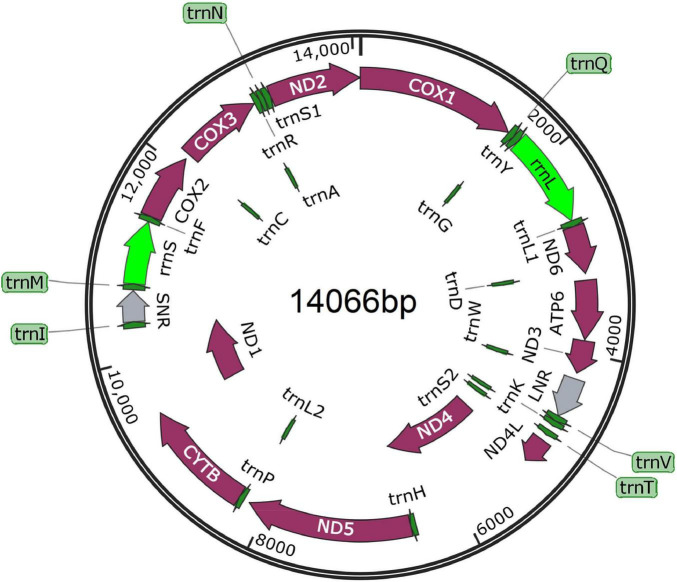
Composition of the complete mitochondrial genome of *Moniliformis* sp. XH-2020. All genes are arranged clockwise with the names of the genes annotated. LNR and SNR represent long non-coding and short non-coding regions.

The complete *mt* genome sequence was manually assembled using the nine overlapping *mt*DNA fragments by joining the 5’ and 3’ ends of two adjacent fragments. A total length of 14,066 bp *mt*DNA was realized after manual assemblage. The 36 genes of the parasite *mt* genome includes 12 PCGs (*cox*1-3, *nad*1-6, *atp*6, *nad*4L and *cyt*b), 22 transfer RNA genes (tRNAs), 2 ribosomal RNA genes (*rrn*L and *rrn*S), and additional 2 non-coding regions (long non-coding region LNR and short non-coding region, SNR), without *atp*8 (Figure 3). The length of *rrn*S was 631 bp, located between *trn*M and *trn*F genes while *rrn*L was 949 bp, and was located between *trn*Y and *trn*L1 genes. SNR (306 bp) was located between *trn*I and *trn*M while LNR (401 bp) was located between *trn*W and *trn*V. The 22 distinct nucleotide sequences ranging from 50 to 66 bp in size for the acanthocephalan were predicted to fold into cloverleaf-like secondary structure of tRNAs with the exception of *trn*S2, which lack a dihydrouridine (DHU) arm (Supplementary Figure 1). Meanwhile, many tRNA secondary structures predicted in this study lack TΨC arm, which is similar to other *mt*DNA studies of acanthocephalans (Steinauer et al., 2005; Gazi et al., 2012, 2015, 2016; Pan and Nie, 2013, 2014; Weber et al., 2013; Muhammad et al., 2019). Because the *mt* genome is a circular type of DNA molecule, the *cox*1 gene was artificially set as the starting point of the entire genome following existing *mt* genome data of acanthocephalans (Gazi et al., 2012; Weber et al., 2013; Wey-Fabrizius et al., 2013).

Named temporarily as *Moniliformis* sp. XH-2020, the nucleotide composition analysis of the complete *mt* genome showed the following: T = 41.2%, C = 8.5%, A = 25.0% and G = 25.3%, with an overall A + T content of 66.2%. The A + T content of the protein-coding *mt* genes was 65.8%, which is a more than that of *O. luehei* (58.8%), slightly more than *P. celatus* (61.2%) and *M. hirudinaceus* (63.9%), but less than that of *Leptorhynchoides thecatus* (71.6%), *Paratenuisentis ambiguus* (67.1%). The results of the nucleotide composition demonstrate the complete *mt* genome of *Moniliformis* sp. XH-2020 is a T-rich *mt*DNA and excessively favors A + T content. The total number of triplet codons of the 12 PCGs in the first position was 3,486 bp, where T account for 33.6%, C 7.7%, A 26.7% and G 32.0% (Supplementary Table 2).

The total length of the 12 PCGs was 10,728 bp, encoding 3,564 amino acids. *Nad*5 gene had the longest sequence (1,641 bp), followed by *cox*1 (1,590 bp), with *nad*4L (261 bp) the shortest. Using the invertebrate mitochondrial code, the relative synonymous codon usage (RSCU) parameter was used to compute codon usage bias, the results demonstrate TTT as the most frequently used codon, with an RSCU value of 1.85, while the codon with the lowest frequency was CTC, with an RSCU of 0.01. The CGC codon was absent (Supplementary Table 3). Among the amino acid sequences, Val amino acid appeared frequently (16.44%) with preference for GTT, GTA and GTG codons, followed by Leu (14.14%) (TTA and TTG) and the least frequent was Gln amino acid (0.61%) (CAG). Initiation/termination codons are shown in Table 2 with more genes utilizing the GTG start codons and TAA stop codons.

**TABLE 2:** Organization of the mitochondrial genome of *Moniliformis* sp. XH-2020.

Sequence	Positions	Length bp	No. of amino acids	Start/stop codons	Intergenic sequences
*cox*1	1-1590	1590	529	GTG/TAG	−55
*trn*G	1535-1589	55			5
*trn*Q	1595-1657	63			0
*trn*Y	1658-1723	66			1
*rrn*L	1725-2673	949			0
*trn*L1	2674-2732	59			0
*nad*6	2733-3209	477	158	GTG/TAG	−47
*trn*D	3163-3216	54			59
*atp*6	3276-3866	591	196	GTG/TAA	10
*nad*3	3877-4203	327	108	GTG/TAA	−2
*trn*W	4202-4262	61			1
LNR	4264-4664	401			0
*trn*V	4665-4723	59			0
*trn*K	4724-4778	55			−1
*trn*E	4778-4832	55			0
*trn*T	4833-4886	54			5
*trn*S2	4892-4950	59			−1
*nad*4L	4950-5210	261	86	GTG/TAG	1
*nad*4	5212-6609	1398	465	GTG/TAG	−131
*trn*H	6479-6532	54			0
*nad*5	6533-8173	1641	546	GTG/TAA	−1
*trn*L2	8173-8232	60			0
*trn*P	8233-8286	54			0
*cytb*	8287-9435	1149	382	GTG/TAG	−20
*nad*1	9416-10312	897	298	ATG/TAA	6
*trn*I	10319-10379	61			0
SNR	10380-10685	306			10
*trn*M	10696-10754	59			0
*rrn*S	10755-11385	631			0
*trn*F	11386-11441	56			0
*cox*2	11442-12110	669	222	ATG/TAA	−2
*trn*C	12109-12162	54			0
*cox*3	12163-12981	819	272	GTG/TAA	−50
*trn*A	12932-12986	55			0
*trn*R	12987-13044	58			0
*trn*N	13045-13094	50			0
*trn*S1	13095-13159	65			0
*nad*2	13160-14065	906	301	ATG/TAA	1

### Phylogenetic Relationships Based on the *cox1* Gene and the Nuclear 18S rDNA Gene

**FIGURE 4:**
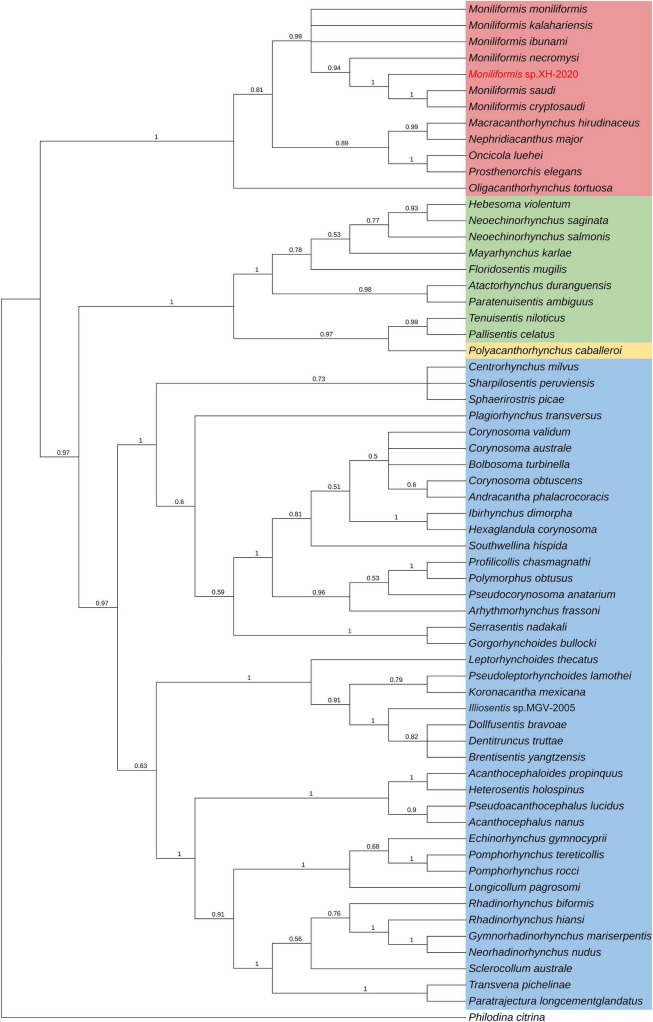
Phylogenetic relationship of *Moniliformis* sp. XH-2020 with other acanthocephalans based on *cox*1 gene by Bayesian inference. Node values are Bayesian posterior probabilities (BPP), the red color indicates the class Archiacanthocephala, the green color indicates the class Eoacanthocephala, the blue color indicates the class Palaeacanthocephala and the yellow color indicates the class Polyacanthocephala.

**FIGURE 5:**
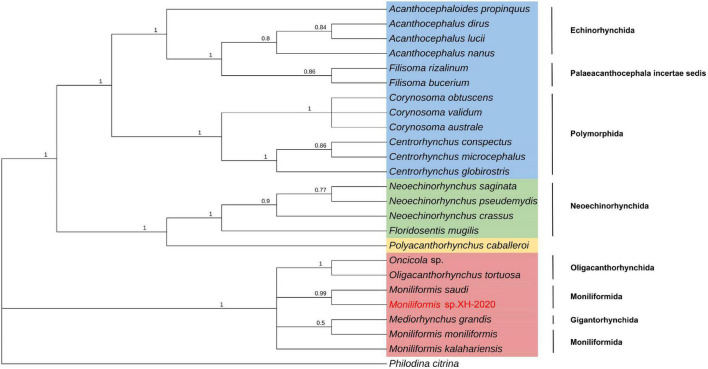
Molecular evolutionary analysis of acanthocephalans based on 18S rDNA gene by Bayesian inference. Node values are Bayesian posterior probabilities (BPP), the red color indicates the class Archiacanthocephala, the green color indicates the class Eoacanthocephala, the blue color indicates the class Palaeacanthocephala and the yellow color indicates the class Polyacanthocephala.

Phylogenetic analysis of *cox*1 gene and 18S rDNA gene inferred by the BI method showed that *Moniliformis* sp. XH-2020 formed a monophyletic group with representative species of the class Archiacanthocephala with strong nodal support values (*cox*1 BPP = 1, 18S rDNA BPP = 1) (Figures 4, 5), and was consistent with the results of ML trees (*cox*1 ML-BP = 100, 18S rDNA ML-BP = 100) (Supplementary Figures 2, 3). Meanwhile, BI tree depicts a monophyletic Archiacanthocephala as the most basal and sister to the other three classes, which sit well with the results of previous investigations (Verweyen et al., 2011; Weber et al., 2013; Gazi et al., 2016; Song et al., 2016; Muhammad et al., 2019). The BI tree of *cox*1 gene showed that *Moniliformis* sp. XH-2020 formed a sister relationship with representative species of the order Moniliformida and forms a monophyletic group (*cox*1 BPP = 0.99) (Figure 4). Similarly, the phylogenetic tree of 18S rDNA gene (including BI and ML trees) also depicted monophyly (Figure 5 and Supplementary Figure 3). Species in the class Archiacanthocephala also formed a monophyletic group both in BI and ML inference of the *cox*1 and 18S rDNA genes (*cox*1 BPP = 1.00, 18S rDNA BPP = 1). In the BI tree of 18S rDNA gene, *Mediorhynchus grandis* in the order Gigantorhynchida formed a clade with *Moniliformis moniliformis* (Figure 5), this relationship is consistent with previous phylogenetic results of small subunits of ribosome DNA (SSU) (Lynggaard et al., 2021).

The class Palaeacanthocephala represented by 40 taxa were divided into two clusters in BI trees (Figures 4, 5). For the phylogenetic results of *cox*1, a cluster was formed by members of the order Polymorphida, Palaeacanthocephala incertae sedis and Echinorhynchida. In this cluster, *Sharpilosentis peruviensis* of the order Palaeacanthocephala incertae sedis and two species of the order Echinorhynchida (*Serrasentis nadakali* and *Gorgorhynchoides bullock*) formed a monophyletic group with members of the order Polymorphida (Figure 4), similar to ML tree output (Supplementary Figure 2). Another paraphyletic cluster was constituted by members of the order Echinorhynchida and Palaeacanthocephala incertae sedis (Figure 4). Moroever, similar observations has also been made (Garcia-Varela and Nadler, 2005; Verweyen et al., 2011; Radwan, 2012). For the class Polyacanthocephala, the representative species *Polyacanthorhynchus caballeroi* formed a monophyletic group with members of class Eoacanthocephala both in BI and ML trees (Figures 4, 5 and Supplementary Figures 2, 3), in contrast to previous morphological classification that placed Polyacanthocephala species within the Palaeacanthocephala (Schmidt and Canaris, 1967; Bullock, 1969; Amin, 1985). The taxonomic status of the class Polyacanthocephala remains controversial due to the classification limitations and unverified species. Particularly, the question of whether it represents a separate class has become a long-held taxonomic issue.

The widespread application of *mt* markers and evidence from previous studies (Verweyen et al., 2011; Martins et al., 2017) suggest that a revisit to the current taxonomic classification is warranted as there seems to be a lack of phylogenetic resolution and consistency between species of certain orders. Case in point, the phylogenetic relationship between species of the class Palaeacanthocephala, and the taxonomic status of the class Polyacanthocephala. Therefore, effective molecular markers and sufficient effective species are urgently needed to reclassify acanthocephalans in the future. In the phylogenetic analysis of species, researchers are increasingly interested in mitochondrial gene markers because of its maternal inheritance, accumulation of spontaneous mutation characteristics, and rate of evolution (Brown et al., 1979; Jia et al., 2012). Compared with *mt* genes, 18S rDNA gene is relatively conserved and is one of the molecular markers commonly used in phylogenetic analysis (Amin et al., 2019). Therefore, the combination of mitochondrial genes and nuclear genes as molecular markers for species classification may provide unique insights into species classification.

### Phylogenetic Relationships Based on the Concatenated Amino Acid Sequences of 12 Protein-Coding Genes and *mt* Gene Order

**FIGURE 6:**
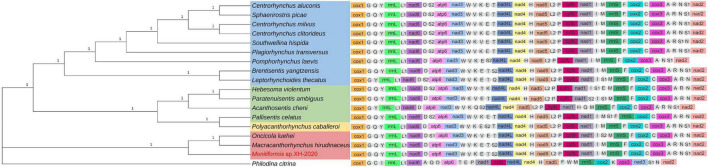
Phylogenetic relationship of *Moniliformis* sp. XH-2020 with other acanthocephalans based on the concatenated amino acid sequences of 12 PCGs by Bayesian inference and *mt* gene order. Node values are Bayesian posterior probabilities (BPP), the red color indicates the class Archiacanthocephala, the green color indicates the class Eoacanthocephala, the blue color indicates the class Palaeacanthocephala, the yellow color indicates the class Polyacanthocephala, the tRNAs are labeled by single-letter code for the corresponding amino acid.

To further verify the phylogeny reconstructed by *cox*1 and 18S rDNA gene, the phylogenetic relationship was inferred by the concatenated amino acid sequences of the 12 PCGs (3,641 characters of the amino acid sequence) of 18 taxa in this study (16 acanthocephalans, 1 rotifera and the current isolate) (Figure 6). In BI and ML trees, the four classes were identified as three major monophyletic clades with strong nodal support values; Palaeacanthocephala BPP = 1.00, ML-BP = 100, Archiacanthocephala BPP = 1.00, ML-BP = 100,

Eoacanthocephala and Polyacanthocephala, BPP = 1.00, ML-BP = 100 (Figure 6 and Supplementary Figure 4). The monophyletic Archiacanthocephala group contained the current isolate *Moniliformis* sp. XH-2020 and formed a sister taxa (BPP = 1.00, ML-BP = 100) with *M. hirudinaceus* and *O. luehei* of the family Oligacanthorhynchidae, and the tree topologies of these two families is consistent with previous study (Gomes et al., 2020).

The class Palaeacanthocephala is the largest acanthocephalan class in species abundance and represetend by three orders. The representative species formed a monophyletic group with high nodal support values (BPP = 1.00, ML-BP = 100), although inconsistent with the *cox*1 and 18S rDNA phyolgentic inference, this unresolved discrepancy is probably a result of the limited number of complete *mt* geneome sequences used for reconstruction due to scarcity of complete *mt* genome of acanthocephalans in GenBank database. Although *Centrorhynchus aluconis*, *C. clitorideus* and *C. milvus* belonged to the same genus *Centrorhynchus* in order Polymorphida, *C. aluconis* alongside *Sphaerirostris picae* of a different genus, formed a clade (BPP = 1.00, ML-BP = 100) and the other two *Centrorhynchus* spp. formed a clade (BPP = 1.00, ML-BP = 100). This result is inconsistent with previous phylogenetic analysis (Gazi et al., 2016; Song et al., 2016), that placed *Southwellina hispida*, *C. aluconis* and *Plagiorhynchus transversus* in a monophyletic group based on the 12 protein-coding genes. Also, three Echinorhynchid species (*Pomphorhynchus laevis*, *Brentisentis yangtzensis* and *L. thecatus*) were not monophyletic, while *B. yangtzensis* and *L. thecatus* formed a well-supported clade (BPP = 1.00 ML-BP = 100). *P. caballeroi* was nested within Eoacanthocephala, forming a clade with *Pallisentis celatus* (BPP = 1.00, ML-BP = 100) and in agreement with the phylogenetic result of *cox*1 and 18S rDNA genes. Nonetheless, due to insufficient species sequence information, the problem of whether both groups are monophyletic or paraphyletic remains a subject of future investigation.

The order of *mt* gene arrangement was relatively consistent with other closely related species which is an important evidence of relatedness. All acanthocephalans with complete *mt* genome information in GenBank, a total of 17 species from all four classes and a *Bdelloidea* species as an outgroup, were selected for comparison of *mt* gene order (Figure 6). The result showed that the sequence of the 12 PCGs and 2 ribosomal RNA genes were identical, while the position of tRNAs varied with species. Three gene blocks (*nad*2*-cox*1*-trn*G, *trn*Y*-rrn*L*-trn*L1*-nad*6 and *nad*4L*-nad*4*-trn*H*-nad*5) were found to be present in the *mt* genomes of all acanthocephalans, and is consistent with the results of previous studies (except for *trn*N*-nad*2*-cox*1*-trn*G) (Gazi et al., 2012, 2015; Figure 6). The *nad*1 and *nad*3 genes in the outgroup *P. chitrina* were located differently from the acanthocephalan, which provides additional evidence supporting the relatedness among acanthocephalan species. The gene order of all Palaeacanthocephala group species were relatively conserved, with a few species demonstrating different tRNA gene positions. In particular, the *mt* gene arrangement of *C. luconis*, *S. picae*, *C. litorideus*, and *C. melvus* were identical, which further supported the phylogenetic analysis demonstrating evolution from the same ancestors. But the *P. laevis* lacks *trn*R, *trn*C and *trn*T genes, and there were translocations of three tRNAs (*trn*Q, *trn*S2, and *trn*S1) between *B. yangtzensis* and *L. thecatus*. Among the three species in the class Archiacanthocephala, *M. hirudinaceus* and *Moniliformis* sp. XH-2020 showed identical genetic order, similar to *B. yangtzensis* of the class Palaeacanthocephala. The tRNA genes of the *O. luehe*, another species of the class Archiacanthocephala, vary greatly in the position of the *mt* genome. There were many differences observed in the *mt* genome order between species of the class Eoacanthocephala in contrast with the phylogenetic results. *P. caballeroi*, the only representative species of the class Polyacanthocephala, has an almost identical gene arrangement as *P. celatus* (except for translocations of two tRNAs [*trn*S1 and *trn*S2]), and is consistent with the sister-relationship shared in the phylogeny which further strengthens the relationship between Eoacanthocephala and Polyacanthocephala. Although gene arrangement in the *mt*DNA can reflect the relationship between species to an extent, the lack of mitochondrial genome data remains a major contributing factor to the taxonomic challenges of acanthocephalans and thus, warrant more studies.

## Conclusion

Besides the genetic evidence and description of a putatively new species of *Monoliformis* in a wild rodent (*Eospalax fontanierii baileyi*) in China, this study provides the complete mitochondrial genome representing the order Moniliformida for the first time which will serve as reference molecular material for the accurate classification of acanthocephalans in the future and in understanding the transmission and host range of members of Moniliformida.

## Data Availability Statement

The datasets presented in this study can be found in online repositories. The names of the repository/repositories and accession number(s) can be found below: https://www.ncbi.nlm.nih.gov/genbank/, OK415026, 18SrDNA gene accession number OM388438.

## Ethics Statement

The animal study was reviewed and approved by all animals were handled in strict accordance with good animal practice according to the Animal Ethics Procedures and Guidelines of the People’s Republic of China, and the study was approved by the Animal Ethics Committee of Lanzhou Veterinary Research Institute, Chinese Academy of Agricultural Sciences (No. LVRIAEC2012-007).

## Author Contributions

G-DD performed the experiments. LL, H-BY, W-ZJ, and B-QF conceived and designed the experiments. L-SZ, Z-LL, S-ZG, and A-MG completed the collection of samples. JO and Y-DW performed the data analyses. All authors contributed to the article and approved the submitted version.

## Conflict of Interest

The authors declare that the research was conducted in the absence of any commercial or financial relationships that could be construed as a potential conflict of interest.

## Publisher’s Note

All claims expressed in this article are solely those of the authors and do not necessarily represent those of their affiliated organizations, or those of the publisher, the editors and the reviewers. Any product that may be evaluated in this article, or claim that may be made by its manufacturer, is not guaranteed or endorsed by the publisher.

## Footnotes

1
https://www.ncbi.nlm.nih.gov/nucleotide/

2
https://chlorobox.mpimp-golm.mpg.de/geseq.html

3
http://130.235.46.10/ARWEN/

4
http://varna.lri.fr/index.php?

5
http://dambe.bio.uottawa.ca/DAMBE/dambe.aspx

6
https://itol.embl.de/

7
http://iqtree.cibiv.univie.ac.at/

## References

[B1] Amin O. M. (1985). “Classification,” in *Biology of the Acanthocephala*, eds Nickol B. B. Crompton D. W. T. (Cambridge, U.K: Cambridge University Press), 27–72.

[B2] Amin O. M. (2013). Classification of the *Acanthocephala*. *Folia Parasitol*. 60 273–305. 10.14411/fp.2013.031 24261131

[B3] Amin O. M. Heckmann R. A. Osama M. Evans R. P. (2016). Morphological and molecular descriptions of *Moniliformis saudi* sp. n. (*Acanthocephala*: *Moniliformidae*) from the desert hedgehog, *Paraechinus aethiopicus* (Ehrenberg) in Saudi Arabia, with a key to species and notes on histopathology. *Folia Parasitol*. 26:2016.014. 10.14411/fp.2016.014 27189420

[B4] Amin O. M. Heckmann R. A. Sharifdini M. Albayati N. Y. (2019). *Moniliformis cryptosaudi* n. sp. (*Acanthocephala*: *Moniliformidae*) from the Long-eared Hedgehog *Hemiechinus auritus* (Gmelin) (Erinaceidae) in Iraq; A Case of Incipient Cryptic Speciation Related to *M. saudi* in Saudi Arabia. *Acta Parasitol*. 64 195–204. 10.2478/s11686-018-00021-9 30666546

[B5] Berenji F. Fata A. Hosseininejad Z. (2007). A case of *Moniliformis moniliformis* (*Acanthocephala*) infection in Iran. *Korean J. Parasitol*. 45 145–148. 10.3347/kjp.2007.45.2.145 17570979 PMC2526305

[B6] Brown W. M. George M. J. Wilson A. C. (1979). Rapid evolution of animal mitochondrial DNA. *Proc. Natl. Acad. Sci. U. S. A*. 76 1967–1971. 10.1073/pnas.76.4.1967 109836 PMC383514

[B7] Bullock W. L. (1969). “Morphological features as tools and as pitfalls in acanthocephalan systematics,” in *Problems in Systematics of Parasites*, ed. Schmidt G. D. (Baltimore, FL: University Park Press), 9–43.

[B8] Capella-Gutiérrez S. Silla-Martínez J. M. Gabaldón T. (2009). trimAl: a tool for automated alignment trimming in large-scale phylogenetic analyses. *Bioinformatics* 25 1972–1973. 10.1093/bioinformatics/btp348 19505945 PMC2712344

[B9] Darty K. Denise A. Ponty Y. (2009). VARNA: Interactive drawing and editing of the RNA secondary structure. *Bioinformatics* 25 1974–1975. 10.1093/bioinformatics/btp250 19398448 PMC2712331

[B10] Garcia-Varela M. Nadler S. A. (2005). Phylogenetic relationships of *Palaeacanthocephala* (*Acanthocephala*) inferred from SSU and LSU rDNA gene sequences. *J. Parasitol*. 91 1401–1409. 10.1645/GE-523R.1 16539024

[B11] Gazi M. Kim J. García-Varela M. Park C. Littlewood D. T. J. Park J. K. (2016). Mitogenomic phylogeny of *Acanthocephala* reveals novel class relationships. *Zool. Scr*. 45 437–454. 10.1111/zsc.12160

[B12] Gazi M. Kim J. Park J. K. (2015). The complete mitochondrial genome sequence of *Southwellina hispida* supports monophyly of *Palaeacanthocephala* (*Acanthocephala*: *Polymorphida*). *Parasitol. Int*. 64 64–68. 10.1016/j.parint.2015.01.009 25656507

[B13] Gazi M. Sultana T. Min G. S. Park Y. C. Garcia-Varela M. Nadler S. A. (2012). The complete mitochondrial genome sequence of *Oncicola luehei* (*Acanthocephala*: *Archiacanthocephala*) and its phylogenetic position within Syndermata. *Parasitol. Int*. 61 307–316. 10.1016/j.parint.2011.12.001 22198415

[B14] Gomes A. P. N. Costa N. A. Gentile R. Vilela R. V. Maldonado A. (2020). Morphological and genetic description of *Moniliformis necromysi* sp. n. (*Archiacanthocephala*) from the wild rodent *Necromys lasiurus* (*Cricetidae*: *Sigmondontinae*) in Brazil. *J. Helminthol*. 94:e138. 10.1017/S0022149X20000188 32188515

[B15] Guerreiro Martins N. B. Del Rosario Robles M. Navone G. T. (2017). A new species of *Moniliformis* from a Sigmodontinae rodent in Patagonia (Argentina). *Parasitol. Res*. 116 2091–2099. 10.1007/s00436-017-5508-9 28585077

[B16] Jia W. Z. Yan H. B. Lou Z. Z. Ni X. W. Dyachenko V. Li H. M. (2012). Mitochondrial genes and genomes support a cryptic species of tapeworm within *Taenia taeniaeformis*. *Acta Trop*. 123 154–163. 10.1016/j.actatropica.2012.04.006 22569565

[B17] Katoh K. Standley D. M. (2013). MAFFT multiple sequence alignment software version 7: improvements in performance and usability. *Mol. Biol. Evol*. 30 772–780. 10.1093/molbev/mst010 23329690 PMC3603318

[B18] Kennedy C. (2006). “Biogeography and distribution,” in *Ecology of the Acanthocephala*, ed. Kennedy C. R. (Cambridge: Cambridge University Press), 28–51. 10.1017/CBO9780511541902

[B19] Khalaf A. K. Swadi B. F. Mahmoudvand H. (2021). Morphological characterization of *Moniliformis moniliformis* isolated from an Iraqi patient. *J. Parasit. Dis*. 45 128–130. 10.1007/s12639-020-01287-5 33746397 PMC7921275

[B20] Khaldi M. Torres J. Samso B. Miquel J. Biche M. Benyettou M. (2012). Endoparasites (helminths and coccidians) in the hedgehogs *Atelerix algirus* and *Paraechinus aethiopicus* from Algeria. *Afr. Zool*. 47 48–54. 10.3377/004.047.0114

[B21] Kozlov D. P. (1977). *Key to the helminths of carnivores in the USSR. Opreditel’ gel’mintov khishchnykh mlekopitayushchikh SSSR.* Moscow, Russia: Nauka, 276.

[B22] Kumar S. Stecher G. Tamura K. (2016). MEGA7: molecular evolutionary genetics analysis version 7.0 for bigger datasets. *Mol. Biol. Evol.* 33, 1870–1874. 10.1093/molbev/msw054 27004904 PMC8210823

[B23] Laslett D. Canback B. (2008). ARWEN: a program to detect tRNA genes in metazoan mitochondrial nucleotide sequences. *Bioinformatics* 24 172–175. 10.1093/bioinformatics/btm573 18033792

[B24] Letunic I. Bork P. (2019). Interactive Tree Of Life (iTOL) v4: recent updates and new developments. *Nucleic Acids Res*. 47 256–259. 10.1093/nar/gkz239 30931475 PMC6602468

[B25] Lynggaard C. García-Prieto L. Guzmán-Cornejo C. García-Varela M. (2021). Description of a new species of *Moniliformis* (*Acanthocephala*: *Moniliformidae*) from *Peromyscus hylocetes* (*Rodentia*: *Cricetidae*) in Mexico. *Parasitol. Int*. 83:102315. 10.1016/j.parint.2021.102315 33677125

[B26] Martins N. B. G. Robles M. D. R. Navone G. T. (2017). A new species of *Moniliformis* from a Sigmodontinae rodent in Patagonia (Argentina). *Parasitol. Res*. 116 2091–2099. 28585077 10.1007/s00436-017-5508-9

[B27] Muhammad N. Suleman, Ma J. Khan M. S. Wu S. S. Zhu X. Q. (2019). Characterization of the complete mitochondrial genome of *Centrorhynchus milvus* (*Acanthocephala*: *Polymorphida*) and its phylogenetic implications. *Infect. Genet. Evol.* 75:e103946. 10.1016/j.meegid.2019.103946 31279002

[B28] Pan T. S. Nie P. (2013). The complete mitochondrial genome of *Pallisentis celatus* (*Acanthocephala*) with phylogenetic analysis of acanthocephalans and rotifers. *Folia Parasitol*. 60 181–191. 10.14411/fp.2013.021 23951925

[B29] Pan T. S. Nie P. (2014). The cloning of the mitochondrial genome of *Hebesoma violentum* (*acanthocephala*) and the phylogenetic analysis of acanthocephalans. *Acta Hydrobiol. Sin*. 38 351–361. 10.7541/2014.50

[B30] Radwan N. A. (2012). Phylogenetic analysis of *Sphaerirostris picae* (*Acanthocephala*: *Centrorhynchidae*) based on large and small subunit ribosomal DNA gene. *Int. J. Parasitol. Res.* 4 106–110.

[B31] Ronquist F. Huelsenbeck J. P. (2003). MrBayes 3: Bayesian phylogenetic inference under mixed models. *Bioinformatics* 19 1572–1574. 10.1093/bioinformatics/btg180 12912839

[B32] Schmidt G. D. (1972). Revision of the class Archiacanthocephala Meyer, 1931 (Phylum Acanthocephala), with emphasis on Oligacanthorhynchidae. *J. Parasitol.* 58, 290–297. 5022866

[B33] Schmidt G. D. Canaris A. G. (1967). *Acanthocephala* from Kenya with descriptions of two new species. *J. Parasitol*. 53 634–637. 10.2307/3276730 6026855

[B34] Song R. Zhang D. Deng S. M. Ding D. M. Liao F. C. Liu L. S. (2016). The complete mitochondrial genome of *Acanthosentis cheni* (*Acanthocephala*: *Quadrigyridae*). *Mitochondrial DNA Part B Resour.* 1 797–798. 10.1080/23802359.2016.1197076 33473631 PMC7799462

[B35] Steinauer M. L. Nickol B. B. Broughton R. Ortí G. (2005). First sequenced mitochondrial genome from the phylum *Acanthocephala* (*Leptorhynchoides thecatus*) and its phylogenetic position within Metazoa. *J. Mol. Evol*. 60 706–715. 10.1007/s00239-004-0159-8 15909226

[B36] Su J. H. Aryal A. Nan Z. B. Ji W. H. (2015). Climate change-induced range expansion of a subterranean rodent: implications for rangeland management in Qinghai-Tibetan Plateau. *PLoS One* 10:e138969. 10.1371/journal.pone.0138969 26406891 PMC4583544

[B37] Su J. H. Hegab I. M. Ji W. H. Nan Z. B. (2018). Function-related drivers of skull morphometric variation and sexual size dimorphism in a subterranean rodent, Plateau Zokor (*Eospalax baileyi*). *Ecol. Evol*. 8 4631–4643. 10.1002/ece3.3986 29760903 PMC5938458

[B38] Tillich M. Lehwark P. Pellizzer T. Ulbricht-Jones E. S. Fischer A. Bock R. (2017). GeSeq - versatile and accurate annotation of organelle genomes. *Nucleic Acids Res*. 45 6–11. 10.1093/nar/gkx391 28486635 PMC5570176

[B39] Trifinopoulos J. Nguyen L. T. von Haeseler A. Minh B. Q. (2016). W-IQ-TREE: a fast online phylogenetic tool for maximum likelihood analysis. *Nucleic Acids Res*. 44 232–235. 10.1093/nar/gkw256 27084950 PMC4987875

[B40] Verweyen L. Klimpel S. Palm H. W. (2011). Molecular phylogeny of the *Acanthocephala* (class *Palaeacanthocephala*) with a paraphyletic assemblage of the orders *Polymorphida* and Echinorhynchida. *PLoS One* 6:e28285. 10.1371/journal.pone.0028285 22163005 PMC3230623

[B41] Weber M. Wey-Fabrizius A. R. Podsiadlowski L. Witek A. Schill R. O. Sugar L. (2013). Phylogenetic analyses of endoparasitic *Acanthocephala* based on mitochondrial genomes suggest secondary loss of sensory organs. *Mol. Phylogenet. Evol*. 66 182–189. 10.1016/j.ympev.2012.09.017 23044398

[B42] Wey-Fabrizius A. R. Podsiadlowski L. Herlyn H. Hankeln T. (2013). Platyzoan mitochondrial genomes. *Mol. Phylogenet. Evol*. 69 365–375. 10.1016/j.ympev.2012.12.015 23274056

[B43] Xia X. H. (2018). DAMBE7: New and improved tools for data analysis in molecular biology and evolution. *Mol. Biol. Evol*. 35 1550–1552. 10.1093/molbev/msy073 29669107 PMC5967572

