# Supplementary material for: Molecular Characterization of a New Moniliformis sp. From a Plateau Zokor (Eospalax fontanierii baileyi) in China
Source: Front Microbiol. 2022 Mar 9;13:806882. doi: 10.3389/fmicb.2022.806882 (PMC8959414; doi:10.3389/fmicb.2022.806882)
Supplement: Supplementary file 1 [file Data_Sheet_1.docx]

# Supplementary Materials

Table S1: Type of reference sequences and their GenBank accession number used in the present study

| Items | Species | Sequence type | GenBank accession No. |
| --- | --- | --- | --- |
| In-group | *Moniliformis ibunami* | *cox*1 | MW115576.1 |
|  | *Moniliformis necromysi* | *cox*1 | MT803593.1 |
|  | *Moniliformis cryptosaudi* | *cox*1 | MH401041.1 |
|  | *Neoechinorhynchus saginata* | *cox*1 | DQ089704.1 |
|  | *Prosthenorchis elegans* | *cox*1 | KT818504.1 |
|  | *Neoechinorhynchus salmonis* | *cox*1 | KF156889.1 |
|  | *Mayarhynchus karlae* | *cox*1 | KY077086.1 |
|  | *Atactorhynchus duranguensis* | *cox*1 | KY077097.1 |
|  | *Tenuisentis niloticus* | *cox*1 | KT970470.1 |
|  | *Sharpilosentis peruviensis* | *cox*1 | KP967562.1 |
|  | *Andracantha phalacrocoracis* | *cox*1 | LC465403.1 |
|  | *Ibirhynchus dimorpha* | *cox*1 | GQ981438.1 |
|  | *Hexaglandula corynosoma* | *cox*1 | EU189488.1 |
|  | *Profilicollis chasmagnathi* | *cox*1 | MT580124.1 |
|  | *Polymorphus obtusus* | *cox*1 | JX442195.1 |
|  | *Pseudocorynosoma anatarium* | *cox*1 | KX688148.1 |
|  | *Arhythmorhynchus frassoni* | *cox*1 | EU189484.1 |
|  | *Serrasentis nadakali* | *cox*1 | KC291713.1 |
|  | *Gorgorhynchoides bullocki* | *cox*1 | DQ089715.1 |
|  | *Pseudoleptorhynchoides lamothei* | *cox*1 | EU090949.1 |
|  | *Koronacantha mexicana* | *cox*1 | DQ089708.1 |
|  | *Illiosentis* sp. MGV-2005 | *cox*1 | DQ089705.1 |
|  | *Dollfusentis bravoae* | *cox*1 | MK294064.1 |
|  | *Dentitruncus truttae* | *cox*1 | JX460903.1 |
|  | *Heterosentis holospinus* | *cox*1 | MN715355.1 |
|  | *Pseudoacanthocephalus lucidus* | *cox*1 | LC100069.1 |
|  | *Echinorhynchus gymnocyprii* | *cox*1 | MT169775.1 |
|  | *Pomphorhynchus tereticollis* | *cox*1 | JQ809452.1 |
|  | *Pomphorhynchus rocci* | *cox*1 | JQ824373.1 |
|  | *Longicollum pagrosomi* | *cox*1 | KY490048.1 |
|  | *Rhadinorhynchus biformis* | *cox*1 | MN692683.1 |
|  | *Rhadinorhynchus hiansi* | *cox*1 | MN203138.1 |
|  | *Gymnorhadinorhynchus mariserpentis* | *cox*1 | MK012667.1 |
|  | *Neorhadinorhynchus nudus* | *cox*1 | MG838935.1 |
|  | *Sclerocollum australe* | *cox*1 | MN692686.1 |
|  | *Transvena pichelinae* | *cox*1 | MN104896.1 |
|  | *Paratrajectura longcementglandatus* | *cox*1 | MK770615.1 |
|  | *Acanthocephalus lucii* | 18S rDNA | AY830152 |
|  | *Acanthocephalus dirus* | 18S rDNA | AY830151 |
|  | *Centrorhynchus globirostris* | 18S rDNA | KM588206 |
|  | *Centrorhynchus microcephalus* | 18S rDNA | AF064813 |
|  | *Centrorhynchus conspectus* | 18S rDNA | U41399 |
|  | *Filisoma bucerium* | 18S rDNA | AF064814 |
|  | *Filisoma rizalinum* | 18S rDNA | JX014229 |
|  | *Mediorhynchus grandis* | 18S rDNA | AF001843 |
|  | *Neoechinorhynchus crassus* | 18S rDNA | KU363974 |
|  | *Neoechinorhynchus pseudemydis* | 18S rDNA | KU363973 |
|  | *Oncicola* sp. | 18S rDNA | AF064818 |
|  | *Acanthosentis cheni* | *mt*DNA | KX108947 |
|  | *Centrorhynchus aluconis* | *mt*DNA | KT592357 |
|  | *Centrorhynchus clitorideus* | *mt*DNA | MT113355 |
|  | *Pomphorhynchus laevis* | *mt*DNA | MN562482 |
|  | *Brentisentis yangtzensis* | *mt*DNA | MK651258 |
|  |  | *cox*1 | MK651258.1 |
|  | *Centrorhynchus milvus* | *mt*DNA | MK922344 |
|  |  | *cox*1 | MK922344.1 |
|  | *Hebesoma violentum* | *mt*DNA | KC415004 |
|  |  | *cox*1 | KF156893.1 |
|  | *Leptorhynchoides thecatus* | *mt*DNA | AY562383 |
|  |  | *cox*1 | DQ089706.1 |
|  | *Macracanthorhynchus hirudinaceus* | *mt*DNA | FR856886 |
|  |  | *cox*1 | LC350021.1 |
|  | *Oncicola luehei* | *mt*DNA | JN710452 |
|  |  | *cox*1 | NC_016754.1 |
|  | *Pallisentis celatus* | *mt*DNA | NC_022921 |
|  |  | *cox*1 | NC_022921.1 |
|  | *Paratenuisentis ambiguus* | *mt*DNA | NC_019807 |
|  |  | *cox*1 | NC_019807.1 |
|  | *Plagiorhynchus transversus* | *mt*DNA | NC_029767 |
|  |  | *cox*1 | NC_029767.1 |
|  | *Sphaerirostris picae* | *mt*DNA | MK471355 |
|  |  | *cox*1 | MK471355.1 |
|  | *Southwellina hispida* | *mt*DNA | NC_026516 |
|  |  | *cox*1 | NC_026516.1 |
|  | *Acanthocephaloides propinquus* | 18S rDNA | AY830149 |
|  |  | *cox*1 | DQ089713.1 |
|  | *Acanthocephalus nanus* | 18S rDNA | LC129889 |
|  |  | *cox*1 | LC100070.1 |
|  | *Corynosoma australe* | 18S rDNA | MK119255 |
|  |  | *cox*1 | MZ920067.1 |
|  | *Corynosoma validum* | 18S rDNA | JX442170 |
|  |  | *cox*1 | JX442193.1 |
|  | *Corynosoma obtuscens* | 18S rDNA | JX442169 |
|  |  | *cox*1 | JX442192.1 |
|  | *Floridosentis mugilis* | 18S rDNA | AF064811 |
|  |  | *cox*1 | MT514243.1 |
|  | *Moniliformis saudi* | 18S rDNA | KU206782 |
|  |  | *cox*1 | KU206783.1 |
|  | *Moniliformis kalahariensis* | 18S rDNA | MH401042 |
|  |  | *cox*1 | MH401040.1 |
|  | *Moniliformis moniliformis* | 18S rDNA | Z19562 |
|  |  | *cox*1 | AF416998.2 |
|  | *Neoechinorhynchus saginata* | 18S rDNA | AY830150 |
|  |  | *cox*1 | DQ089704.1 |
|  | *Oligacanthorhynchus tortuosa* | 18S rDNA | AF064817 |
|  |  | *cox*1 | AF416999.2 |
|  | *Polyacanthorhynchus caballeroi* | 18S rDNA | AF388660 |
|  |  | *mt*DNA | KT592358 |
|  |  | *cox*1 | DQ089724.1 |
| Out-group | *Philodina citrina* | 18S rDNA | JX494740 |
|  |  | *mt*DNA | FR856884 |
|  |  | *cox*1 | FR856884.1 |
| Host | *Eospalax baileyi* | *cox*1 | NC018098 |

Table S2: The base composition of the mitochondrial genome

| Nucleotide | T (%) | C (%) | A (%) | G (%) | A+T (%) | G+C (%) | Size (bp) |
| --- | --- | --- | --- | --- | --- | --- | --- |
| Entire *mt* sequence | 41.2 | 8.5 | 25.0 | 25.3 | 66.2 | 33.8 | 14,066 |
| Protein coding-genes | 42.7 | 8.1 | 23.1 | 26.1 | 65.8 | 34.2 | 10,728 |
| ribosomal RNA genes | 36.1 | 9.3 | 32.5 | 22.1 | 68.6 | 31.4 | 1,580 |
| transfer RNA genes | 38.1 | 10.6 | 29.9 | 21.4 | 68.0 | 32.0 | 1,274 |
| Non-coding region | 34.8 | 8.8 | 31.8 | 24.6 | 66.6 | 33.4 | 707 |

Table S3: Mitochondrial genome codon usage

| Codon | Count | RSCU | Codon | Count | RSCU | Codon | Count | RSCU | Codon | Count | RSCU |
| --- | --- | --- | --- | --- | --- | --- | --- | --- | --- | --- | --- |
| TTT (F) | 231 | 1.85 | TCT (S2) | 125 | 2.53 | TAT (Y) | 153 | 2.49 | TGT (C) | 32 | 1.78 |
| TTC (F) | 19 | 0.15 | TCC (S2) | 8 | 0.16 | TAC (Y) | 24 | 0.39 | TGC (C) | 4 | 0.22 |
| TTA (L2) | 217 | 2.58 | TCA (S2) | 57 | 1.15 | TAA (*) | 7 | 0.11 | TGA (W) | 40 | 0.75 |
| TTG (L2) | 193 | 2.29 | TCG (S2) | 20 | 0.41 | TAG (*) | 5 | 1 | TGG (W) | 67 | 1.25 |
| CTT (L1) | 42 | 0.5 | CCT (P) | 41 | 2.31 | CAT (H) | 43 | 1.76 | CGT (R) | 15 | 1.71 |
| CTC (L1) | 1 | 0.01 | CCC (P) | 7 | 0.39 | CAC (H) | 6 | 0.24 | CGC (R) | 0 | 0 |
| CTA (L1) | 23 | 0.27 | CCA (P) | 14 | 0.79 | CAA (Q) | 8 | 0.73 | CGA (R) | 14 | 1.6 |
| CTG (L1) | 29 | 0.34 | CCG (P) | 9 | 0.51 | CAG (Q) | 14 | 1.27 | CGG (R) | 6 | 0.69 |
| ATT (I) | 201 | 1.62 | ACT (T) | 45 | 2.37 | AAT (N) | 74 | 1.98 | AGT (S1) | 61 | 1.24 |
| ATC (I) | 21 | 0.17 | ACC (T) | 6 | 0.32 | AAC (N) | 6 | 0.16 | AGC (S1) | 4 | 0.08 |
| ATA (I) | 151 | 1.21 | ACA (T) | 16 | 0.84 | AAA (K) | 32 | 0.86 | AGA (S1) | 46 | 0.93 |
| ATG (M) | 171 | 1 | ACG (T) | 9 | 0.47 | AAG (K) | 46 | 1 | AGG (S1) | 74 | 1.5 |
| GTT (V) | 213 | 1.44 | GCU (A) | 50 | 2.13 | GAT (D) | 70 | 1.92 | GGT (G) | 91 | 1.27 |
| GTC (V) | 19 | 0.13 | GCC (A) | 8 | 0.34 | GAC (D) | 3 | 0.08 | GGC (G) | 12 | 0.17 |
| GTA (V) | 187 | 1.26 | GCA (A) | 22 | 0.94 | GAA (E) | 32 | 0.68 | GGA (G) | 62 | 0.87 |
| GTG (V) | 173 | 1.17 | GCG (A) | 14 | 0.6 | GAG (E) | 62 | 1.32 | GGG (G) | 121 | 1.69 |


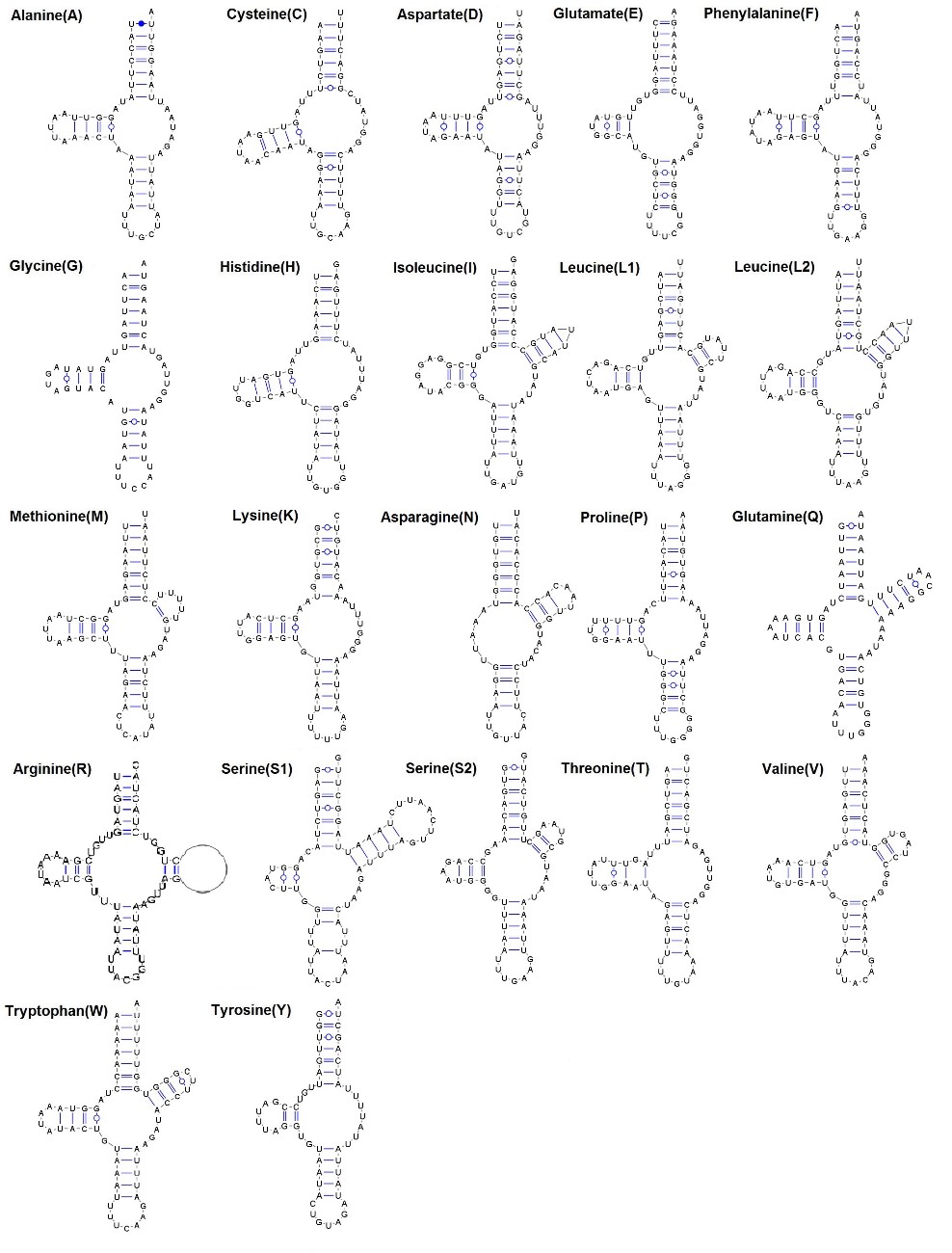


Figure S1: The predicted secondary structures of 22 tRNAs of *Moniliformis* sp. XH-2020 mitochondrial DNA.


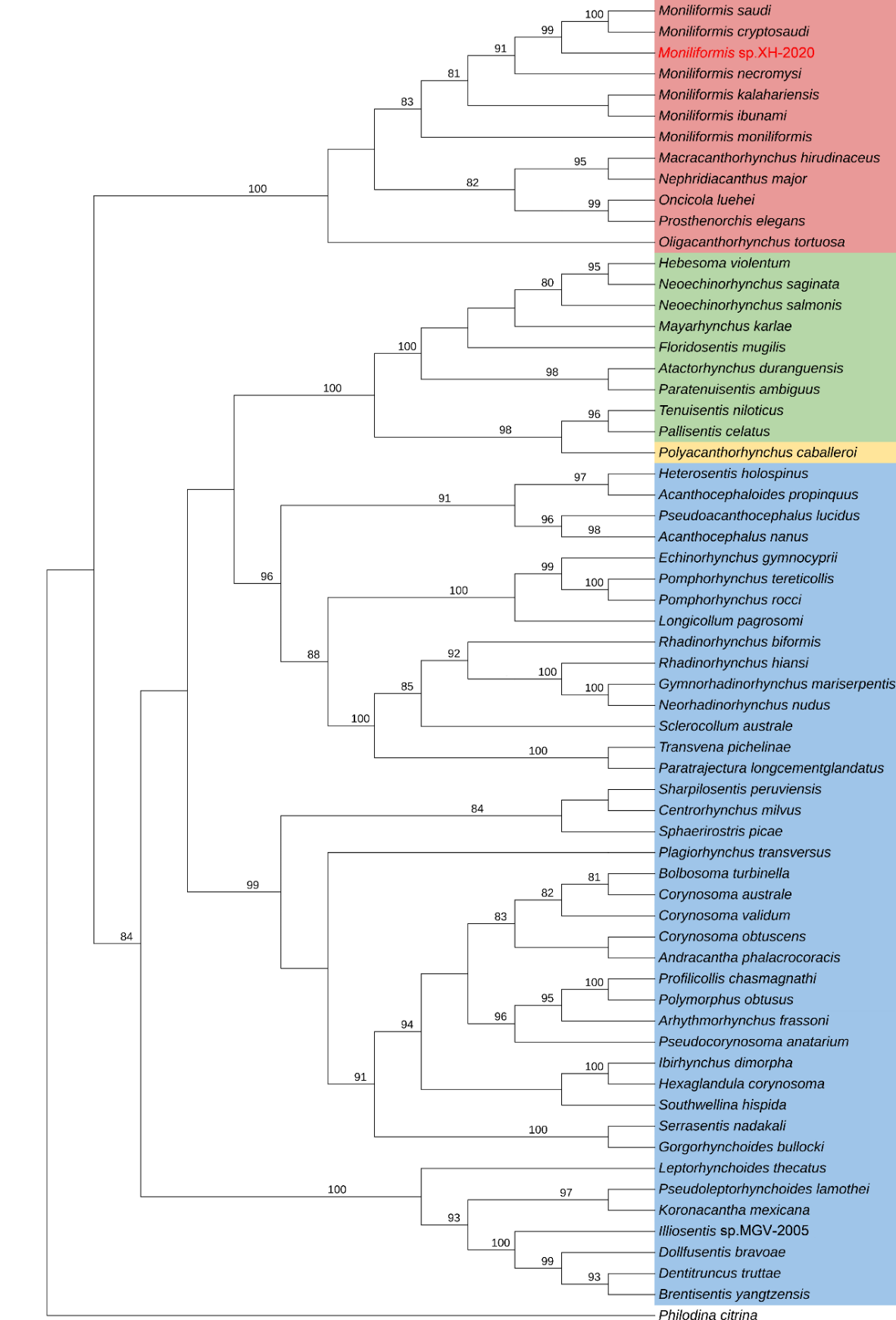


Figure S2: Phylogenetic relationship of *Moniliformis* sp. XH-2020 with other acanthocephalans based on the *cox*1 gene by Maximum-Likelihood (ML) (*Philodina citrina* as an outgroup).


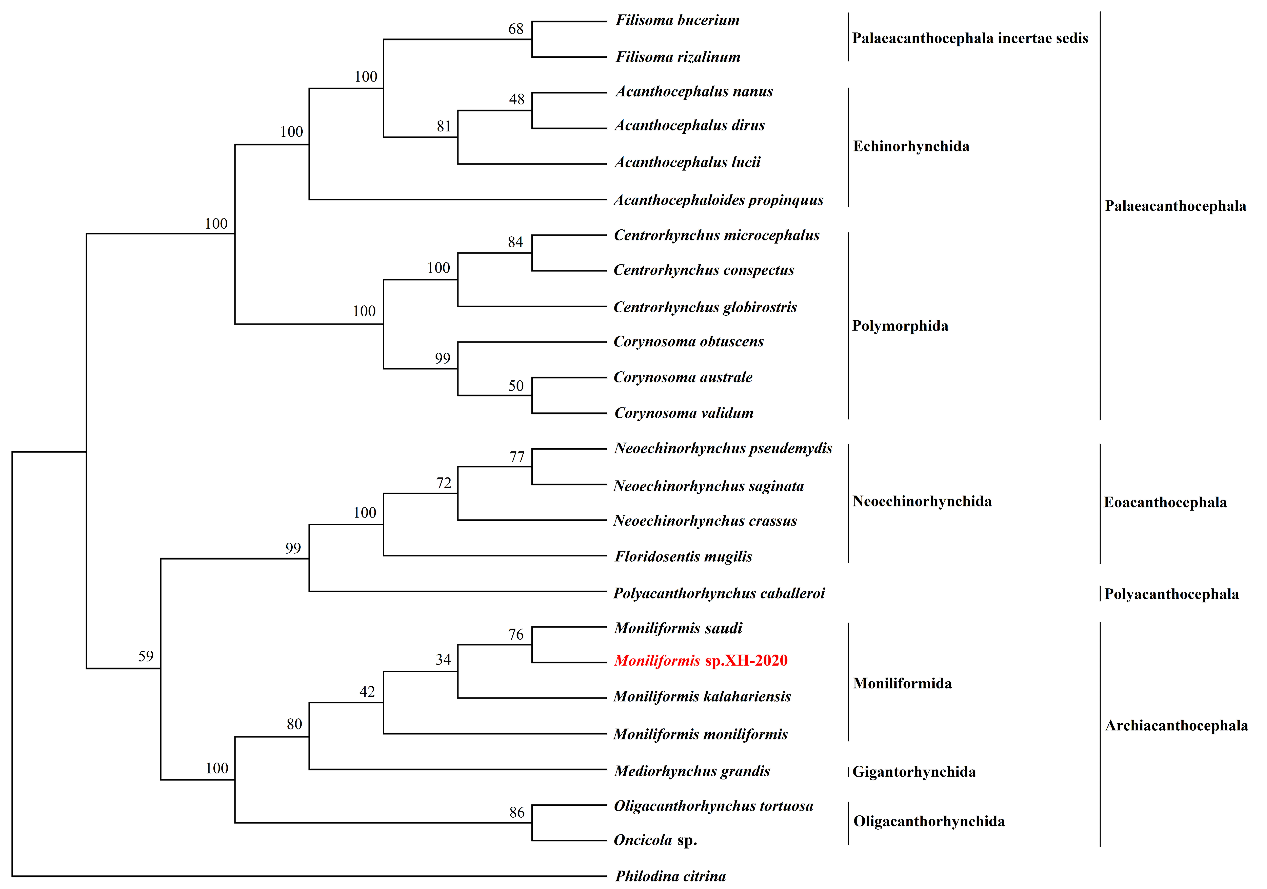


Figure S3: Phylogenetic relationship of *Moniliformis* sp. XH-2020 with other acanthocephalans based on the 18S rDNA of 23 representatives by Maximum-Likelihood (ML).


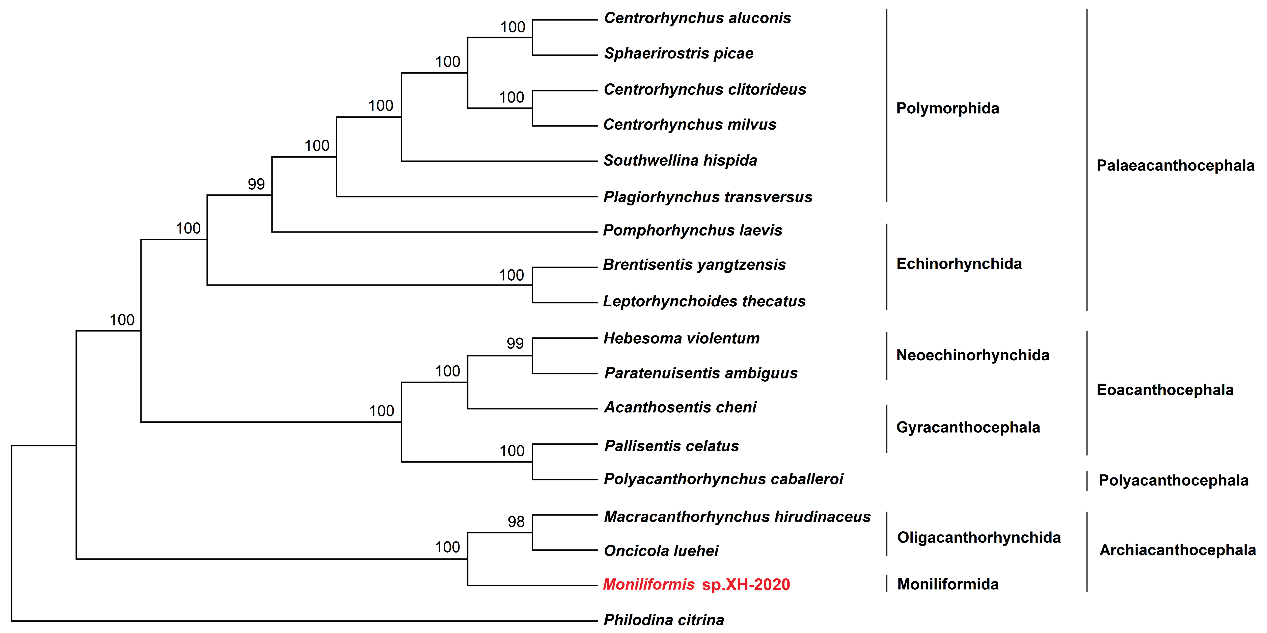


Figure S4: Phylogenetic relationship of *Moniliformis* sp. XH-2020 with other acanthocephalans based on the concatenated amino acid sequences of 12 PCGs of 17 representatives by Maximum-Likelihood (ML).
